# Generalized lymphatic anomaly involving the pleura and bone in an older male: A case report

**DOI:** 10.1016/j.rmcr.2023.101961

**Published:** 2023-12-09

**Authors:** Atsushi Yanagisawa, Akihiro Tamiya, Takayuki Takimoto, Hiromitsu Sumikawa

**Affiliations:** a Department of Internal Medicine, National Hospital Organization Kinki-Chuo Chest Medical Center, Kitaku Nagasone-cho 1180, Sakai City, Osaka, 591-8555, Japan; b Department of Radiology, National Hospital Organization Kinki-Chuo Chest Medical Center, Kitaku Nagasone-cho 1180, Sakai City, Osaka, 591-8555, Japan

**Keywords:** Generalized lymphatic anomaly (GLA), Multiple bone lesion, Pleural lesion, Adult

## Abstract

Generalized lymphatic anomaly (GLA) is a congenital malformation of the lymphatic vessels that is often diagnosed in early childhood. Owing to the rarity and heterogeneity of its clinical course, GLA is frequently misdiagnosed, especially in adults. A 67-year-old man was incidentally found to have bone and pleural lesions. Multiple bone lesions detected on magnetic resonance images were mistaken for malignancy, and pathological evaluation led to the diagnosis of GLA. GLA should be considered in the differential diagnosis of multiple bone lesions, and a proactive biopsy to confirm the diagnosis may help avoid unnecessary invasive procedures.

## Highlights

• GLA is frequently misdiagnosed, especially in adults.
• Bone and pleural lesions in a 67-year-old man were mistaken for malignancy on MRI.
• Pathological evaluation led to the diagnosis of GLA.
• GLA should be considered in the differential diagnosis of multiple bone lesions.

## 1 Introduction

Generalized lymphatic anomaly (GLA) is a rare congenital disorder involving the aberrant overgrowth of lymphatic vessels [1] that usually occurs in early childhood but may not be detected until adulthood in some cases [2]. GLA can occur in any tissue in which lymphatic vessels normally exist, but typically involves the skeletal and pulmonary systems [3] and multiple organs, making it difficult to distinguish it from a malignancy [4]. Owing to its rarity, most evidence on GLA is derived from case reports or case series, and little is known about the clinical features of this disease.

Herein we report the case of an older male patient with GLA presenting both pleural and bone involvement.

## 2 Case presentation

**Fig. 1:**
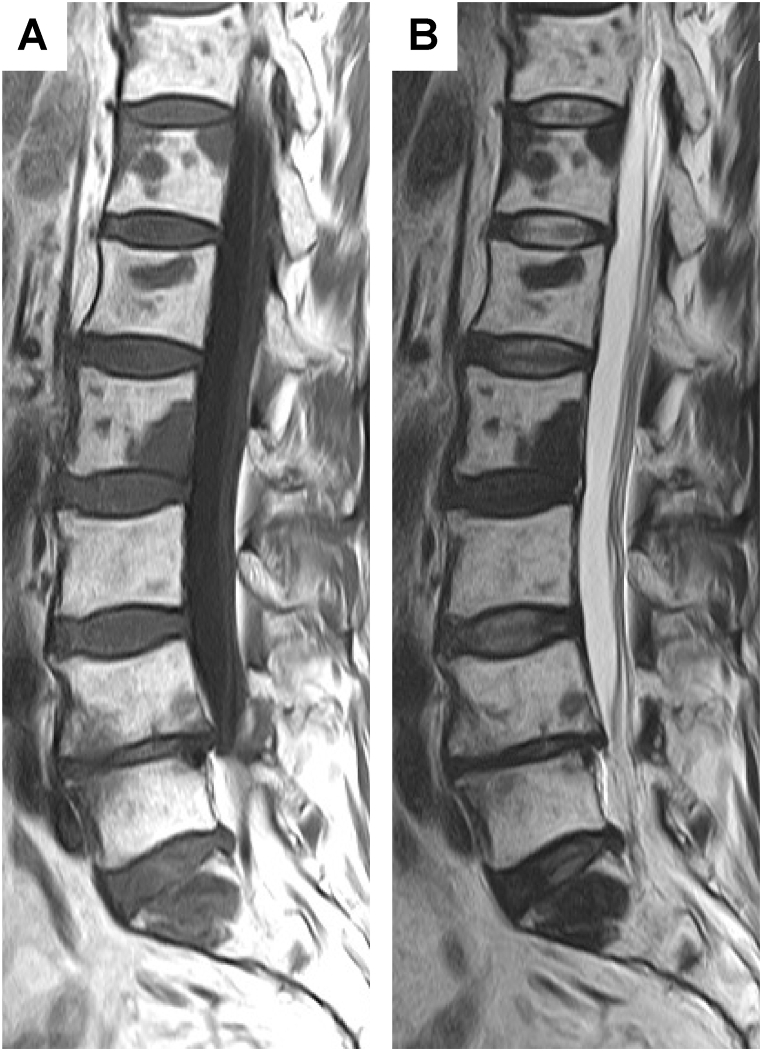
Sagittal magnetic resonance image of the thoracic spine shows multiple vertebral body lesions. T1-weighted (A) and T2-weighted (B) images show multiple hypointense masses.

**Fig. 2:**
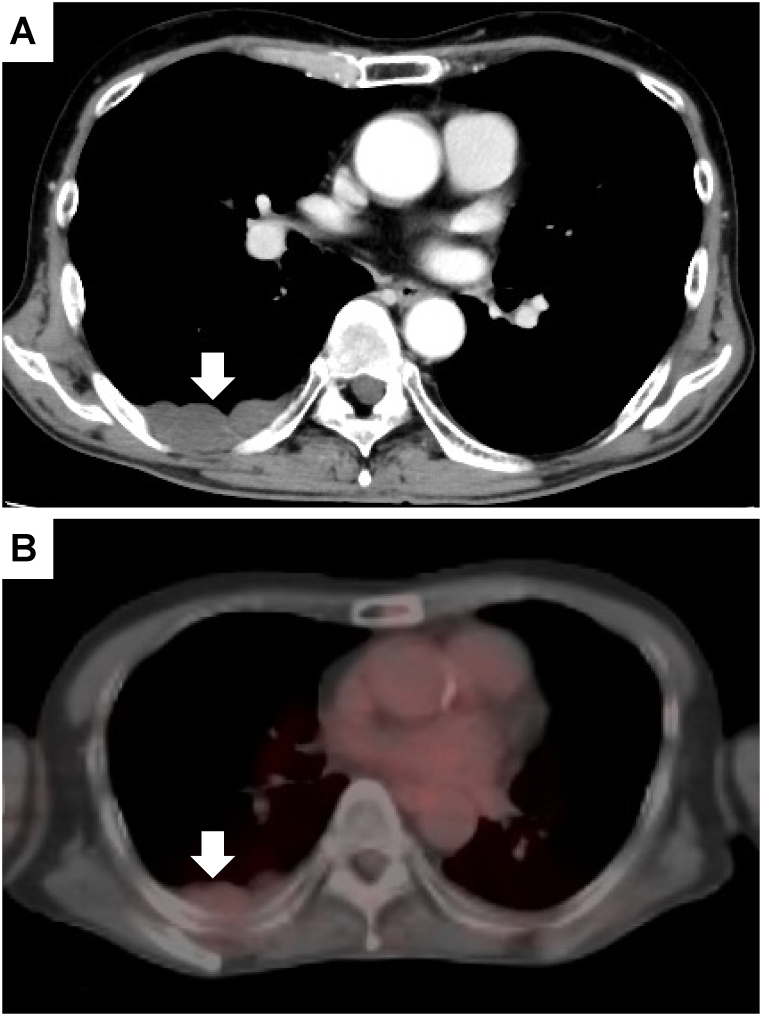
Contrast-enhanced computed tomography (CT) scan reveals a hypoattenuating lesion on the patient's right dorsal pleura (A), which shows no fluorodeoxyglucose uptake on positron emission tomography CT (B). The white arrow indicates the lesion.

A 67-year-old man presented to his general practitioner with a chief complaint of slowly progressive bilateral mild numbness in his lower legs. The patient had a smoking history of 47 pack-years and habitual alcohol consumption. No neurological abnormalities were observed during a physical examination, and blood tests revealed no abnormal values, including those of tumor markers. Subsequent spinal magnetic resonance imaging (MRI) was performed to identify the cause of the numbness, which revealed multiple lesions with a decreased signal on T1-weighted and T2-weighted images throughout the thoracic and lumbar spine (Fig. 1). There was no evidence of spinal canal stenosis that could have caused numbness. Since bone metastasis of a malignant tumor was suspected, we performed whole-body contrast-enhanced computed tomography (CT). A hypoattenuating lesion in the right dorsal pleura was detected (Fig. 2A), but there were no abnormal shadows in the lungs and no suspected tumor masses in other organs. Fluorine-18-fluorodeoxyglucose (^18^F-FDG) positron emission tomography (PET)-CT showed no remarkable ^18^F-FDG uptake in either the pleural lesion (Fig. 2B) or the bone lesions (not shown).

**Fig. 3:**
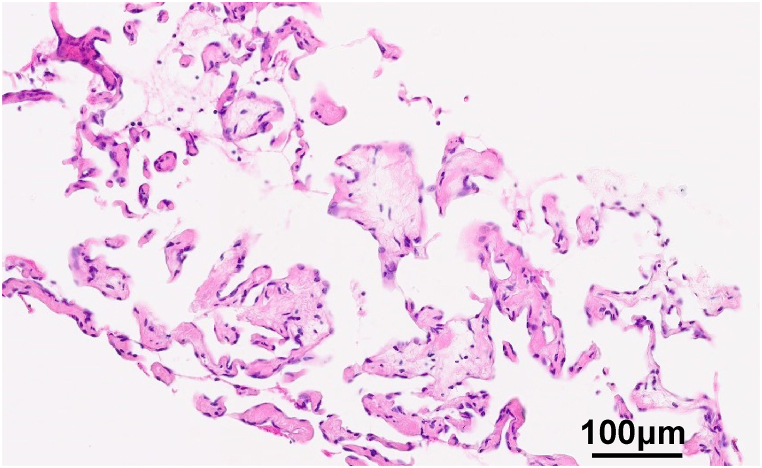
Histology of the right pleural lesion. Hematoxylin-eosin staining of the specimen revealed a marked proliferation of dilated lymphatic structures, which were consistent with the generalized lymphatic anomaly.

To confirm the diagnosis, we performed a CT-guided biopsy of the right pleural mass one month after the patient's initial visit. Hematoxylin-eosin staining of the biopsy specimen revealed a marked proliferation of complex anastomosing lymphatic canals (Fig. 3). No evidence of malignancy was observed in the biopsied tissues. Based on radiological and pathological findings, the pleural and multiple bone lesions were classified as GLA.

We instructed the patient to stop drinking alcohol because we considered the bilateral numbness in his lower legs to be a symptom of alcoholic neuropathy. Numbness resolved shortly after these instructions were given. The patient was monitored conservatively without any medication for his asymptomatic GLA. There were no changes in symptoms or the size of the pleural lesion on CT imaging 5 years after the initial diagnosis.

## 3 Discussion

We report the case of an older male with incidentally detected GLA with pleural and bone involvement. Since the patient was of a susceptible age for malignancy, we initially suspected that the multiple bone masses on MRI were metastatic. However, a biopsy of the pleural lesions confirmed the diagnosis of GLA. We subsequently confirmed that the pleural lesion did not enlarge over time without treatment.

The patient visited our hospital complaining of numbness; however, no neurological abnormalities were found, and the numbness quickly improved when the patient stopped drinking alcohol. Considering the patient's clinical course, there was no correlation between the numbness and GLA lesions. We suspected that these lesions existed before symptom onset and were discovered incidentally.

GLA is a rare non-neoplastic congenital disease characterized by diffuse or multicentric proliferation of dilated lymphatic vessels [1]. Vascular endothelial factor receptor 3 has been suggested to play an important role in GLA development; however, the exact pathophysiology of GLA has not been clearly defined [5]. GLA often involves multiple tissues and has been reported to involve the skeleton, lung, mediastinum, spleen, liver, kidney, colon, and retroperitoneum [3]. Owing to the rarity of the disease and its unique course in each patient, GLA is often misdiagnosed, and a definitive diagnosis is often delayed [5,6]. GLA usually presents in childhood but can be diagnosed in adults [2] and is very difficult to differentiate from malignancy because of the similarities in appearance on imaging [[7], [8], [9]]. Although the prognosis is poor in some pediatric patients, the prognosis in adult patients is generally favorable [7].

About 75 % of patients with GLA have bone lesions, which often occur in multiples and are varied in size, making them difficult to differentiate from bone tumors [4]. Liu et al. reported a case of GLA diagnosis in a 35-year-old woman following an incidental detection of multiple bone lesions and a hypodense soft tissue mass occupying the anterior mediastinum on CT, which were difficult to distinguish from malignancy as in our case [7]. This report highlights the need for definitive diagnosis by proactive biopsy to avoid the invasive procedures required in cases of malignancy [7]. As imaging tests such as CT and MRI become more widely available to asymptomatic individuals, the number of GLA cases incidentally found in adults, which might have been previously missed, could also increase. This case illustrates the importance of increased awareness among clinicians, because GLA can be an incidental discovery, even at an advanced age. Given that it is difficult to diagnose GLA by imaging alone, proactive biopsy of the lesion is important for diagnosis and determining the course of treatment.

Common bone lesions in GLA show osteolytic changes as hypointense and hyperintense on T1-weighted and T2-weighted MRI [[10], [11], [12]], respectively, whereas the bone lesions in our case appeared as hypointense T2-weighted signals and were considered osteoblastic changes. In adults, GLA involving the bones has been reported to show a less aggressive osteolytic pattern than that of the known features in children [4]. It is possible that GLA bone lesions that do not enlarge found in adults, as in this case, tend to appear as low-intensity masses on T2-weighted MRI; however, this has not been adequately studied and remains uncertain. Further studies are required to confirm this hypothesis.

In this case, PET-CT showed no uptake in the GLA lesion. A previous case study of GLA indicated that PET-CT revealed FDG uptake within a lesion, making it difficult to distinguish it from a malignancy [8]. Conversely, another case study found no FDG uptake on PET-CT, indicating a decreased possibility of malignancy [9]. Studies have reported a lower detection rate using PET-CT for osteoblastic bone metastases than for osteolytic bone metastases [13]. These findings suggest that GLA bone lesions with osteoblastic changes show weaker uptake on PET-CT than those with osteolytic changes. Bone scintigraphy, which provides superior detection of bone lesions with osteoblastic changes, was not performed in this case. There are few reports on nuclear medicine scans for GLA, and further case accumulation is desirable.

There was no pleural effusion or lung field shadow observed despite pleural involvement in our patient's case. GLA often causes chylothorax because of the spontaneous rupture of abnormally dilated lymphatic vessels [14]. However, in this case, there was no evidence of pleural fluid accumulation, even after a biopsy of the pleural lesion. In addition, when GLA lesions are detected in the pleura, diffuse pulmonary lymphangiomatosis (DPL) is likely to occur [15]; however, we observed no evidence suggesting DPL, such as abnormal thickening of the interlobular septal wall or other shadows in the lungs. To the best of our knowledge, there have been no previous reports of GLA with pleural involvement but without chylothorax or lung field involvement.

There is no definitive cure for GLA, and all current treatments are supportive and symptomatic. Although some drugs such as sirolimus and bevacizumab have been reported to be effective in the treatment of GLA [16,17], observation alone is appropriate if the lesions are not enlarging or are asymptomatic [7]. In our case, the patient had no symptoms caused by GLA; thus, he was discharged without any medication. Five years after the diagnosis, the patient was still fine.

## 4 Conclusions

In the present case, GLA was difficult to differentiate radiographically from malignant bone metastases, and a tissue biopsy was required to determine an accurate diagnosis. These findings suggest that GLA should be included in the differential diagnosis of multiple bone lesions, regardless of patient age.

## Ethical approval

The patient's data were anonymized.

This study was approved by the Institutional Review Board at the National Hospital Organization Kinki-Chuo Chest Medical Center (approval number: 2022-109).

## Authorship statement

All authors met the International Committee of Medical Journal Editors authorship criteria. A.Y. wrote the manuscript. All authors contributed to the editing of the manuscript and approved the final version of the manuscript.

## Declaration of competing interest

We know of no conflicts of interest associated with this publication, and there has been no financial support for this work that could have influenced its outcome. As Corresponding Author, I confirm that the document has been read and approved for submission by all the named authors.
